# Low heart rate variability relates to the progression of gastric cancer

**DOI:** 10.1186/s12957-018-1348-z

**Published:** 2018-03-07

**Authors:** Songjie Hu, Jie Lou, Youping Zhang, Ping Chen

**Affiliations:** Department of Surgery, Ningbo No. 2 Hospital, Yongfeng Road, Ningbo, Zhejiang Province 315010 China

**Keywords:** Gastric cancer, Heart rate variability, Vagal nerve activity, C-reactive protein, Stage

## Abstract

**Background:**

Gastric cancer (GC) is one of the most common malignant gastrointestinal tumors with the high morbidity and mortality, affecting the quality of human life. This study aimed to identify the role of heart rate variability (HRV) in patients with GC.

**Methods:**

From January 2010 to June 2014, 383 consecutive patients diagnosed with GC were enrolled in this study. Clinical and pathological information from each patient were retrospectively recorded. HRV, including standard deviation of normal-to-normal RR intervals (SDNN) and root mean square of successive differences (RMSSD), were measured by electrocardiography.

**Results:**

The results showed that the SDNN and RMSSD in GC patients were 19.02 ± 13.58 ms and 21.64 ± 17.57 ms, respectively. HRV decreased with advanced clinical stage (*P* < 0.0001). HRV correlated with tumor size, tumor infiltration, lymph node metastasis and distant metastasis (*P* < 0.001); however, no correlation with tumor site and metastasis severity was found (*P* > 0.05). C-reactive protein (CRP) was higher in the low HRV group than that in high HRV group (*P* = 0.008).

**Conclusions:**

GC patients showed a lower HRV that was correlated with tumor stage. HRV decreased with tumor progression, which may be related to a mechanism involving vagal nerve excitement inhibiting the inflammatory reaction.

## Background

Gastric cancer (GC) is one of the most common malignant gastrointestinal tumors. About 600,000 persons die of GC each year worldwide [1]. GC incidence is high in China, with an occurrence rate of approximately 20 per 100,000 persons, accounting for half of all the GC cases globally [2]. It ranks second in mortality rate among all the common cancers [3]. Early detection remains urgent for improving GC prognosis.

Heart rate variability (HRV) is a parameter measuring the time variation of the sinus rhythm, reflecting the regulatory effects of the nervous system and humoral factors on the sinoatrial node [4]. HRV measurement is a simple and reliable method for quantitatively evaluating the status and variations of the autonomic nervous system. Currently, RR interval data are usually recorded using the dynamic electrocardiogram and are used to observe HRV over a long period of time [5]. Studies across the world focus on the application of HRV data in treatment and prognosis of coronary heart disease and diabetes [6–8]. Recently, researchers have devoted themselves to the role of the vagus nerve activity, which is indexed by HRV in cancer prognosis [9]. A role for HRV in the prognosis of breast cancer [10] and colorectal cancer [11] has been reported. However, studies on HRV in GC patients are rare.

The standard deviation of normal-to-normal RR intervals (SDNN) and root mean square of successive differences (RMSSD) are two indices of HRV. The present study was therefore conducted to explore the role of HRV in GC prognosis by observing the correlation between SDNN, RMSSD, and clinical pathological parameters. Inflammation-related mechanisms were also discussed.

## Methods

### Ethics

The research protocol was reviewed and approved by the Research Ethics Committee of Ningbo No. 2 Hospital. All participants gave written consent for their clinical information to be used for scientific research.

### Study design

#### Patients

From January 2010 to June 2014, 383 consecutive patients diagnosed with GC were enrolled, including 274 males and 109 females, with a mean age of 60.72 years (range 60.72 ± 11.82 years). All patients were confirmed to have GC by pathological examination. Patients who had a history of cardiac disease, such as coronary heart disease, arrhythmia, and hyperthyroidism, were excluded.

Clinical and pathological data for each patient were recorded according to the NCCN Guidelines for Gastric Cancer (NCCN, 2014). Preoperative ultrasonic endoscopy and postoperative pathological examination were used to determine maximal tumor diameter, Borrmann classification, and invasion depth. The cancer histological type, invasion depth, lymph node involvement, and peritoneal and liver metastasis were recorded according to the seventh AJCC TNM staging system. Papillary adenocarcinoma and tubular adenocarcinoma were defined as differentiated cancer [12, 13]. Poorly differentiated adenocarcinoma, signet-ring cell carcinoma, and mucinous adenocarcinoma were defined as undifferentiated cancer.

#### HRV measurement

Electrocardiography (ECG) monitoring was conducted for all patients for 20 min by an ECG with a Burdick Vision Holter Recorder (Cardiac Science, Bothell, Washington, USA). The standard deviation of all normal-to-normal intervals (SDNNs) and root mean square successive differences (RMSSDs) were measured for time-domain analysis of HRV by the same researcher.

#### Statistical analysis

Clinical and pathological data for all the GC patients were collected and analyzed by PASW 18.0 statistics software for Windows (SPSS Inc., Chicago, IL, USA). Continuous data were expressed as the mean ± SD. A chi-square test was performed to analyze the correlation between parameters. Analysis of variance (ANOVA) was conducted to compare the differences among groups, with *P* value < 0.05 defined as a significant difference.

## Results

### HRV distribution among GC patients

The SDNN and RMSSD in the GC patients were 19.02 ± 13.58 ms and 21.64 ± 17.57 ms, respectively. As shown in Table 1, the SDNNs and RMSSDs did not differ significantly by sex or age. However, patients presenting with Borrmann I-II tumors had SDNN and RMSSD (24.14 and 27.84 ms), significantly higher than those of Borrmann III-IV tumor patients (18.84 and 20.83 ms) (*P* = 0.008 and 0.013, respectively), which indicated that a significant decrease in HRV occurred in patients with infiltrated GC.

**Table 1:** Correlation of clinical factors with HRV in gastric cancer

	SDNN (ms)	*P* value	RMSSD (ms)	*P* value
Sex		0.507		0.052
Male (*n* = 274)	19.30 ± 13.48		22.74 ± 19.04	
Female (*n* = 109)	18.28 ± 13.85		18.88 ± 12.78	
Age (years)		0.177		0.298
< 65 (*n* = 229)	19.78 ± 14.17		22.41 ± 17.59	
≥ 65 (*n* = 154)	17.87 ± 12.59		20.50 ± 17.51	
Borrmann type		0.008		0.013
I and II (*n* = 44)	24.14 ± 18.89		27.84 ± 28.47	
III and V (*n* = 339)	18.34 ± 12.61		20.83 ± 15.49	
Tumor location		0.436		0.364
Cardia (*n* = 37)	20.90 ± 17.64		24.82 ± 27.83	
Body (*n* = 115)	19.01 ± 11.84		21.48 ± 13.88	
Antrum (*n* = 229)	18.82 ± 13.68		21.35 ± 17.14	
Diffuse (*n* = 2)	5.54 ± 3.37		4.48 ± 0.31	
Tumor size		0.001		0.002
≤ 2 cm (*n* = 30)	27.47 ± 16.40		32.29 ± 20.99	
2–5 cm (*n* = 229)	18.65 ± 14.40	0.502^a^	20.95 ± 19.13	0.771^a^
> 5 cm (*n* = 124)	17.63 ± 10.23		20.33 ± 12.06	

*SDNN* standard deviation of all normal-to-normal intervals, *RMSSD* root mean square successive difference

^a^Compared with the group of 2–5 cm tumor size

The SDNNs and RMSSDs among patients with tumors in the cardia, stomach body, and pylorus were not significantly different (*P* = 0.436 and 0.364, respectively), indicating that HRV in GC patients was not correlated with tumor site. However, tumor size had a significant impact on HRV: the SDNNs and RMSSDs were significantly different if the tumor was < 2 cm compared with those > 2 cm (*P* < 0.05), but if the tumor size was 2–5 cm or > 5 cm, there were no significant differences (*P* = 0.502 and 0.771, respectively).

### Correlation between HRV and GC pathological parameters

As shown in Table 2, the SDNN and RMSSD in patients with differentiated GCs were 20.89 and 24.05, respectively. This was not significantly different from those with undifferentiated tumors (*P* = 0.109 and 0.103, respectively), indicating that HRV changes in GC patients were not correlated with cancer tissue differentiation. However, as the clinical stage advanced, both the SDNN and RMSSD displayed a decreasing trend (*P* < 0.0001). Furthermore, patients with lymph node metastasis had significantly lower SDNNs and RMSSDs than those without lymph node metastasis (*P* < 0.0001). However, the severity of metastasis was not significantly related to the SDNNs or RMSSDs (*P* = 0.363 and 0.153, respectively). Similarly, patients with distant metastasis had significantly lower SDNNs and RMSSDs than those without distant metastasis (*P* = 0.004 and 0.021, respectively).

**Table 2:** Correlation of pathological parameters with HRV in gastric cancer

	SDNN (ms)	*P* value	RMSSD (ms)	*P* value
Histological type		0.109		0.113
Differentiated (*n* = 99)	20.89 ± 17.56		24.05 ± 23.09	
Undifferentiated (*n* = 283)	18.35 ± 11.84		20.80 ± 15.13	
T		0.000		0.000
T1 (*n* = 20)	29.96 ± 21.96		35.75 ± 26.28	
T2 (*n* = 57)	24.96 ± 16.50		27.41 ± 22.07	
T3 (*n* = 280)	17.43 ± 11.86		19.79 ± 15.58	
T4 (*n* = 26)	14.51 ± 6.64		17.98 ± 10.03	
LN involvement		0.000		0.000
Negative (*n* = 78)	29.23 ± 19.64		31.09 ± 26.23	
Positive (*n* = 305)	16.40 ± 10.01	0.363	19.22 ± 13.58	0.153
N1 (*n* = 116)	17.40 ± 10.45		21.00 ± 15.67	
N2 (*n* = 165)	15.67 ± 9.82		17.85 ± 12.41	
N3 (*n* = 24)	16.52 ± 9.10		20.07 ± 9.10	
Metastasis		0.004		0.021
No (*n* = 348)	19.64 ± 19.94		22.30 ± 18.09	
Yes (*n* = 35)	12.70 ± 6.51		15.10 ± 8.77	
TNM stage		0.000		0.000
I + II (*n* = 100)	28.24 ± 18.07		30.89 ± 25.35	
III + IV (*n* = 283)	15.75 ± 9.69		18.38 ± 13.30	
Liver metastasis		0.085		0.100
No (*n* = 374)	19.20 ± 13.66		21.87 ± 17.68	
Yes (*n* = 9)	11.30 ± 5.67		12.12 ± 7.15	
Peritoneal metastasis		0.006		0.025
No (*n* = 352)	19.58 ± 13.88		22.23 ± 18.01	
Yes (*n* = 31)	12.56 ± 6.72		14.88 ± 9.02	

*SDNN* standard deviation of all normal-to-normal intervals, *RMSSD* root mean square successive difference, *LN* lymph node

If a SDNN of 20 ms was chosen as the threshold, low HRV (SDNN < 20 ms) significantly correlated with tumor size, T stage, and lymph node involvement (Table 3, *P* < 0.0001). These results indicated that HRV decreased significantly as the cancer advanced, and HRV in GC patients was significantly correlated with clinical stage and the presence or absence of lymphatic/distant metastasis.

**Table 3:** Correlation of clinical factors with HRV in gastric cancer

	SDNN < 20 ms	SDNN ≥20 ms	*P* value
Tumor size			0.000
≤ 2 cm	13	20	
2–5 cm	157	69	
> 5 cm	86	38	
T stage			0.000
T1–T2	38	39	
T3–T4	218	38	
LN involvement			0.000
Negative	34	44	
Positive	222	83	
TNM stage			0.000
I + II	43	57	
III + IV	213	70	

*SDNN* standard deviation of all normal-to-normal intervals, *RMSSD* root mean square successive difference, *LN* lymph node

### Correlation between HRV and C-reactive protein

C-reaction protein (CRP) in the serum of GC patients was determined by a Beckman DxC600 biochemical analyzer (Beckman Coulter, Fullerton, USA) according to previously reported protocols [14]. CRP in patients with high HRV was significantly lower than in those with low HRV (Table 4, 5.92 ± 9.10 vs. 8.06 ± 9.06; *P* = 0.031), indicating that active inflammation may be ongoing in patients with inhibited HRV.

**Table 4:** The correlation of CRP with HRV and tumor stage of gastric cancer

	CRP (mg/L)	*P* value
SDNN < 20 ms (*n* = 256)	8.06 ± 9.06	0.031
SDNN ≥20 ms (*n* = 126)	5.92 ± 9.10	

*SDNN* standard deviation of all normal-to-normal intervals, *RMSSD* root mean square successive difference, *CRP* C-reaction protein

## Discussion

This study observed the HRV in 308 consecutive GC patients. HRV refers to the speed of the heartbeat or the time-related variations in R-R intervals and reflects the regulation of the cardiovascular system by the autonomic nervous system and subsequent responses [15]. It is one of the major approaches for investigating of cardiac autonomic nervous system function [16]. The SDNN is a time-domain measurement, reflecting overall sympathetic and vagal activity, while RMSSD mainly reflects sympathetic activity [17]. The present study focused on changes of SDNN and RMSSD in GC patients at different stages to explore the prognostic role of HRV. The results indicated that the SDNN and RMSSD decreased with the progression of GC.

Tumor growth can be promoted in many ways: (1) free radicals produced by local inflammation, promoting oxidative stress, can stimulate tumor growth; (2) oxidative stress causes DNA damage in normal cells and impedes subsequent repair mechanisms, which ultimately leads to error accumulation in cancer-related genes; and (3) sympathetic neurotransmitters, such as norepinephrine, can stimulate tumor metastasis and progression. Vagal nerve stimulation, however, can inhibit the release of sympathetic neurotransmitters [18]. Gidron et al. proposed that vagal nerve stimulation controlled and reduced tumor progression [9]. Mid-late-stage tumors are often accompanied by damage to autonomic nerves, and HRV decrease is a symptom of this in patients with late-stage tumors [19]. Clinical studies have shown that HRV, an index of vagal nerve activity [9], might be used to predict severity and prognosis in multiple cancers, including liver [20], breast [21], and colon cancers [11]. However, the correlation between HRV and GC has never been reported to our knowledge. The results for 383 GC patients in our study showed that the SDNN and RMSSD were 19.02 ± 13.58 ms and 21.64 ± 17.57 ms, respectively. These were significantly lower than those observed in the healthy Chinese population, which were 141 ± 39 ms and 39.0 ± 15.0 ms (*P* < 0.0001), respectively [22], suggesting that GC patients presented with vagal nerve damage, consistent with the data obtained from other studies.

The present study compared tumors from different sites and different clinical stages and found that HRV in GC patients was related to tumor size, infiltration depth, and whether or not lymphatic metastasis or distant organ metastasis had occurred. HRV was correlated with tumor size. However, if the tumors were greater than 2 cm, HRV did not decrease with increasing tumor size. The results indicated that damage to the vagal nerves in GC patients was primarily related to tumor spread. Similarly, patients with stage III or IV GC had significantly lower HRV than those with stage I or II, indicating that as the GC progressed, HRV decreased. The results might be related to the lymphatic or distant metastasis of the cancer, subsequent systemic changes in metabolism and immune system, and the emotional depression in patients with late-stage GC. All these factors might cause imbalanced regulation of the autonomic nervous system along the nervous-humoral-adrenal axis [23]. Altogether, these data showed that HRV could serve as a factor to evaluate the staging and prognosis of GC patients. In the present study, age and gender of the patient did not have a significant impact on HRV, consistent with the results from multi-center HRV analysis in China [24].

One of the mechanisms by which vagal nerves limit tumor progression is inhibition of the inflammatory reaction and associated free radicals [25]. Studies have shown that decreased HRV is related to an increase in CRP [26], a biomarker for inflammation reactions. If a SDNN of 20 ms was chosen as the threshold, GC patients with higher HRV also demonstrated significantly higher CRP when compared with lower HRV patients. These results indicated that as GC progresses, the function of the vagal nerve system becomes damaged, resulting in a loss of control of the inflammatory responses resulting from secondary tumor development and further acceleration of tumor progression.

## Conclusions

Our study shows that GC patients have significantly lower HRV when compared with the normal healthy control population, and the decrease in HRV correlates with tumor size and clinical stage. Taken together, these data further confirm from a different perspective that HRV decrease in GC progression may be related to an imbalanced inflammatory response. Therefore, stimulation of sympathetic activity may inhibit tumor progression.
